# Author Correction: Whole-muscle fat analysis identifies distal muscle end as disease initiation site in facioscapulohumeral muscular dystrophy

**DOI:** 10.1038/s43856-023-00236-6

**Published:** 2023-01-12

**Authors:** Linda Heskamp, Augustin Ogier, David Bendahan, Arend Heerschap

**Affiliations:** 1 Department of Medical Imaging/Radiology, Radboud university medical center, Nijmegen, The Netherlands; 2 Aix Marseille Univ, CNRS, CRMBM, Marseille, France

**Keywords:** Neuromuscular disease, Prognostic markers

Correction to: *Communications Medicine* 10.1038/s43856-022-00217-1, published online 01 December 2022.

There was a mistake in the title of Figure 6 of this article. The title previously read ‘Typical examples of muscles in facioscapulohumeral muscular dystrophy (FSHD) patients not showing a decreasing proximo-distal fat infiltration profile’. However, the correct title is ‘Typical examples of muscles in facioscapulohumeral muscular dystrophy (FSHD) patients not showing a decreasing distal-to-proximal fat infiltration profile’. This has now been corrected in the HTML and PDF versions of the article.

