# Supplementary material for: Whole-muscle fat analysis identifies distal muscle end as disease initiation site in facioscapulohumeral muscular dystrophy
Source: Commun Med (Lond). 2022 Dec 1;2:155. doi: 10.1038/s43856-022-00217-1 (PMC9712512; doi:10.1038/s43856-022-00217-1)
Supplement: Supplementary file 4 — Supplementary Information [file 43856_2022_217_MOESM4_ESM.pdf]

Supplementary Information

Whole-muscle fat analysis identifies distal muscle end as disease initiation site in facioscapulohumeral muscular dystrophy

Authors

Linda Heskamp[1], Augustin Ogier[2], David Bendahan[2], Arend Heerschap[1]

[1]Department of Medical Imaging/Radiology, Radboud university medical center, Nijmegen, The Netherlands

[2]Aix Marseille Univ, CNRS, CRMBM, Marseille, France

Supplementary note

Fat fraction change normalized to residual muscle mass

The change in fat fraction is generally calculated over the whole muscle, including both fat and healthy muscle tissue. However, the part of the muscle that is already fully fat replaced cannot be replaced by more fat. To measure the true muscle fat replacement, the change in fat fraction was therefore also calculated by normalization to the residual muscle mass using the following equation

$$Normalized\ fat\ fraction = fat\ fraction\ change \times (1 - baseline\ fat\ fraction)$$

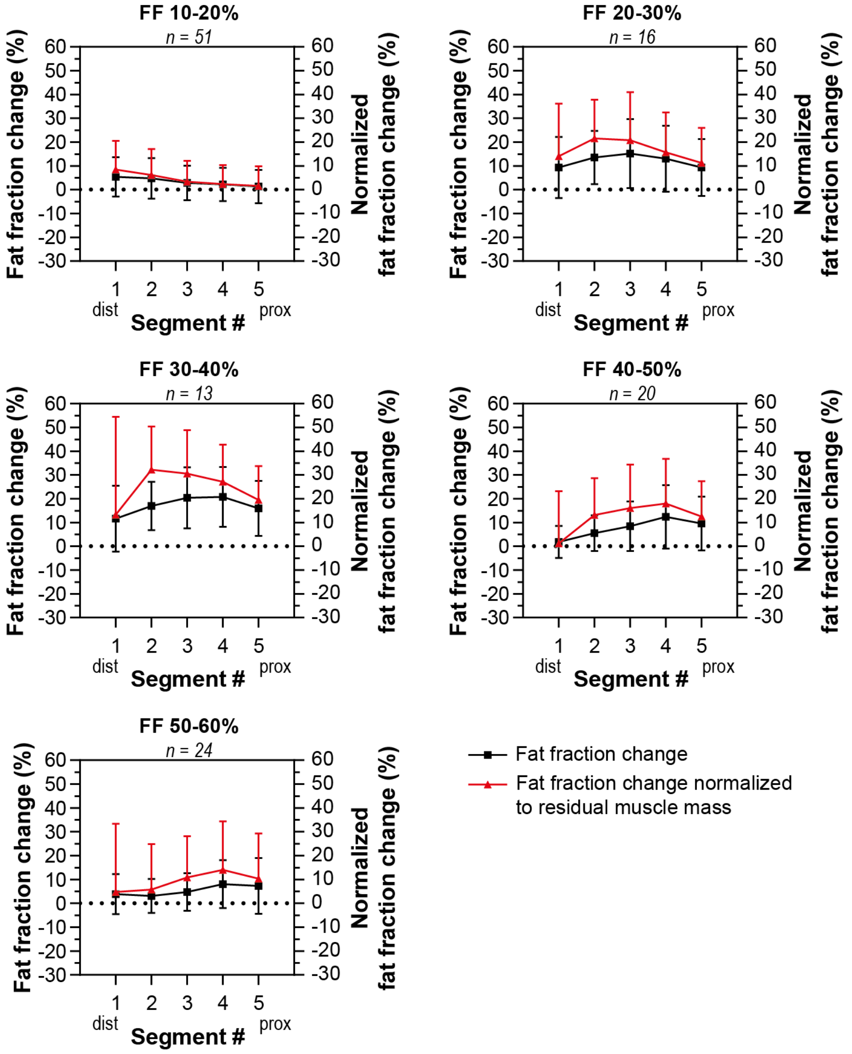

Supplemental figure S1: Progression of fat infiltration along the distal-proximal muscle axis in FSHD patients normalized to residual muscle mass for intermediate fat infiltrated muscles. The fat fraction change normalized to residual muscle mass (red lines) are larger and more strongly accentuate the wave-like pattern of fat infiltration from distal to proximal compared to the fat fraction change as calculated in the standard way (black). The shape of the red lines demonstrate that the segment with the largest increase in fat fraction moves from distal to proximal in this comparison of muscle groups with baseline levels between 10% fat and 60% fat. Data are presented as mean ± SD.

Supplementary Tables

Supplementary Table 1: Follow-up duration, baseline fat fraction, and change in fat fraction for each facioscapulohumeral muscular dystrophy (FSHD) patient.

	FSHD patient number									Average over all patients	
	**#1**	**#2**	**#3**	**#4**	**#5**	**#6**	**#7**	**#8**	**#9**		
**Follow-up duration (years)**	3.75	3.75	3.67	3.83	3.67	3.5	3.42				
**Baseline fat fraction (%)**										**Mean ± SD**	
Lower extremity	8.7	41.4	46.8	23.1	19.4	51.7	52.4	26.9	18.5	32.1 ± 16.2	
Upper legs	9.1	43.3	52.8	31.6	23.9	59.2	56.4	28.6	18.9	36.0 ± 17.8	
Anterior compartments	6.1	45.5	49.3	12.3	10.0	51.3	50.4	11.8	7.9	27.2 ± 21.0	
Posterior compartments	17.3	43.9	65.5	75.5	49.0	80.6	79.6	62.1	45.6	57.7 ± 20.7	
Medial compartments	8.8	36.2	53.3	47.8	43.5	66.1	53.2	32.6	15.6	39.7 ± 18.5	
Lower legs	8.0	35.7	36.5	10.6	11.7	36.6	44.4	23.5	17.8	25.0 ± 13.6	
Anterior compartments	9.3	13.8	55.0	40.2	12.4	38.0	36.8	31.8	11.1	27.6 ± 16.4	
Posterior compartments	7.1	39.5	36.7	8.5	10.3	38.4	53.4	22.2	18.9	26.1 ± 16.5	
Lateral compartments	13.3	42.2	14.0	8.3	18.2	19.7	14.6	23.3	18.4	19.1 ± 9.7	
**Change in fat fraction (%)**										**Mean ± SD**	**Mean ± SD per year**
Lower extremity	0.3	17.0	5.9	5.3	2.0	3.2	-0.6			4.7 ± 5.9	1.3 ± 1.6
Upper legs	0.5	18.4	4.7	7.1	1.4	1.9	-1.0			4.7 ± 6.6	1.3 ± 1.8
Anterior compartments	-0.6	12.8	3.3	5.3	-1.6	2.2	-1.4			2.8 ± 5.1	0.8 ± 1.4
Posterior compartments	2.5	20.5	6.8	1.6	6.7	-1.8	-2.2			4.9 ± 7.6	1.3 ± 2.1
Medial compartments	1.0	30.5	6.2	15.3	9.5	3.1	0.8			9.5 ± 10.6	2.5 ± 2.8
Lower legs	-0.1	12.5	8.1	1.6	2.1	4.1	0.1			4.1 ± 4.7	1.1 ± 1.3
Anterior compartments	1.6	0.3	2.2	-0.4	3.4	3.3	-2.0			1.2 ± 2.0	0.3 ± 0.6
Posterior compartments	0.1	15.4	9.8	2.3	2.2	3.8	0.7			4.9 ± 5.6	1.3 ± 1.5
Lateral compartments	-4.5	14.0	3.2	-2.0	0.5	6.2	-1.7			2.2 6.3	0.6 ± 1.7

Fat fractions were calculated for individual muscles and subsequently averaged to calculate weighted composite scores for the lower extremity, upper leg, lower leg, and the muscle groups, both left and right leg averaged. Standard deviations for weighted composite scores in individual patients are not presented as some muscle groups consisted of only one or two muscles. Upper leg: anterior compartment (rectus femoris, vastii muscles, and sartorius), posterior compartment (biceps femoris, semitendinosus, and semimembranosus), medial compartment (adductor magnus, adductor longus, and gracilis). Lower leg: anterior compartment (tibialis anterior and extensor digitorum longus), posterior compartment (gastrocnemius, soleus, tibialis posterior, flexor hallucis longus, flexor digitorum longus, and popliteus), lateral compartment (peroneus).

Supplemental Table S2: Muscles with an overall baseline fat fraction between 10-18% that show similar fat fractions along the whole muscle length, indicating normal fat content for these muscles.

	FSHD patient #	Muscle	side	Baseline fat fraction
1	#1	Peroneus	Left	13.8
2	#2	Tibialis Anterior	Left	10.9
3	#3	Flexor Digitorum Longus	Right	11.7
4	#5	Popliteus	Left	10.9
5	#5	Extensor Digitorum Longus	Right	13.1
6	#5	Popliteus	Right	12.9
7	#6	Peroneus	Left	14.7
8	#6	Tibialis Posterior	Left	13.4
9	#6	Flexor Hallucis Longus	Left	14.0
10	#6	Tibialis Posterior	Right	16.5
11	#6	Flexor Hallucis Longus	Right	16.7
12	#7	Peroneus	Left	14.7
13	#7	Peroneus	Right	14.5
14	#8	Tibialis Posterior	Left	10.6
15	#8	Flexor Digitorum Longus	Left	10.6
16	#8	Gastrocnemius Lateralis	Left	15.6
17	#8	Flexor Hallucis Longus	Left	11.3
18	#8	Popliteus	Left	16.0
19	#8	Extensor Digitorum Longus	Right	12.2
20	#8	Tibialis Posterior	Right	11.0
21	#8	Flexor Digitorum Longus	Right	10.2
22	#8	Gastrocnemius Lateralis	Right	12.7
23	#8	Flexor Hallucis Longus	Right	14.0
24	#8	Biceps Femoris Short Head	Right	10.8
25	#9	Extensor Digitorum Longus	Right	10.0
26	#9	Peroneus	Right	17.8
27	#9	Biceps Femoris Short Head	Right	14.2
28	#9	Adductor Magnus	Right	17.7

Supplemental Table S3: Muscles that do not show a distal to proximal declining pattern of fat infiltration along the muscle length. Their overall fat fraction levels at baseline and type of fat infiltration pattern are indicated.

	FSHD patient #	Muscle	side	Baseline fat fraction	Type of fat infiltration pattern
1	#1	Tibialis Anterior	Right	12.1	U-shape gradient
2	#1	Peroneus	Right	12.8	True counter-gradient; higher fat fractions proximally
3	#1	Semimembranosus	Right	50.2	Medial fat bulk
4	#2	Biceps Femoris Short Head	Left	30.3	Homogeneous elevated fat fraction
5	#2	Adductor Longus	Right	57.4	Medial fat bulk
6	#3	Rectus Femoris	Left	57.5	True counter-gradient; higher fat fractions proximally
7	#3	Vastus Medialis	Left	58.4	True counter-gradient, higher fat fractions proximally
8	#3	Biceps Femoris Short Head	Left	41.8	Homogeneous elevated fat fraction
9	#3	Tibialis Posterior	Right	15.9	U-shape gradient
10	#4	Extensor Digitorum Longus	Left	44.2	Homogeneous elevated fat fraction
11	#4	Biceps Femoris Short Head	Left	58.5	Homogeneous elevated fat fraction
12	#4	Gracilis	Left	32.5	Medial fat bulk
13	#4	Adductor Magnus	Left	58.4	U-shape gradient
14	#4	Extensor Digitorum Longus	Right	43.1	Homogeneous elevated fat fraction
15	#4	Vastus Medialis	Right	12.9	Medial fat bulk
16	#5	Extensor Digitorum Longus	Left	16.8	True counter-gradient; higher fat fractions proximally
17	#5	Adductor Magnus	Left	52.1	Medial fat bulk
18	#6	Extensor Digitorum Longus	Left	57.7	Medial fat bulk
19	#6	Flexor Digitorum Longus	Left	14.9	Medial fat bulk
20	#6	Tibialis Anterior	Right	17.6	U-shape gradient
21	#6	Peroneus	Right	24.1	Homogeneous elevated fat fraction
22	#6	Vastus Medialis	Right	55.5	Medial fat bulk
23	#7	Tibialis Anterior	Left	59.4	U-shape gradient
24	#7	Rectus Femoris	Left	24.7	Medial fat bulk
25	#9	Tibialis Anterior	Left	10.7	U-shape gradient
26	#9	Adductor Magnus	Left	25.9	Homogeneous elevated fat fraction
27	#9	Tibialis Anterior	Right	13.5	U-shape gradient
28	#9	Rectus Femoris	Right	38.7	Medial fat bulk
