# Supplementary material for: Whole-muscle fat analysis identifies distal muscle end as disease initiation site in facioscapulohumeral muscular dystrophy
Source: Commun Med (Lond). 2022 Dec 1;2:155. doi: 10.1038/s43856-022-00217-1 (PMC9712512; doi:10.1038/s43856-022-00217-1)
Supplement: Supplementary file 5 — Description of Additional Supplementary Files [file 43856_2022_217_MOESM5_ESM.pdf]

Description of Additional Supplementary Files

Filename: Supplementary Data 1

Description: Source data for the figures published in this manuscript. This includes baseline fat fraction and change in fat fraction per participant at the muscle level, the five-segment level and slice level.

Filename: Supplementary Data 2

Description: All post-processed data generated or analysed during this study at the participant level, muscle level, five-segment level and slice level.
