# Supplementary material for: Whole-muscle fat analysis identifies distal muscle end as disease initiation site in facioscapulohumeral muscular dystrophy
Source: Commun Med (Lond). 2022 Dec 1;2:155. doi: 10.1038/s43856-022-00217-1 (PMC9712512; doi:10.1038/s43856-022-00217-1)
Supplement: Supplementary file 6 — Reporting Summary [file 43856_2022_217_MOESM6_ESM.pdf]

nature portfolio

Corresponding author(s): Linda Heskamp

Last updated by author(s): Nov 2, 2022

Reporting Summary

Nature Portfolio wishes to improve the reproducibility of the work that we publish. This form provides structure for consistency and transparency in reporting. For further information on Nature Portfolio policies, see our Editorial Policies and the Editorial Policy Checklist.

Statistics

For all statistical analyses, confirm that the following items are present in the figure legend, table legend, main text, or Methods section.

n/a	Confirmed	
☐	☒	The exact sample size (*n*) for each experimental group/condition, given as a discrete number and unit of measurement
☐	☒	A statement on whether measurements were taken from distinct samples or whether the same sample was measured repeatedly
☐	☒	The statistical test(s) used AND whether they are one- or two-sided<br>*Only common tests should be described solely by name; describe more complex techniques in the Methods section.*
☒	☐	A description of all covariates tested
☐	☒	A description of any assumptions or corrections, such as tests of normality and adjustment for multiple comparisons
☐	☒	A full description of the statistical parameters including central tendency (e.g. means) or other basic estimates (e.g. regression coefficient) AND variation (e.g. standard deviation) or associated estimates of uncertainty (e.g. confidence intervals)
☐	☒	For null hypothesis testing, the test statistic (e.g. *F*, *t*, *r*) with confidence intervals, effect sizes, degrees of freedom and *P* value noted<br>*Give P values as exact values whenever suitable.*
☒	☐	For Bayesian analysis, information on the choice of priors and Markov chain Monte Carlo settings
☒	☐	For hierarchical and complex designs, identification of the appropriate level for tests and full reporting of outcomes
☒	☐	Estimates of effect sizes (e.g. Cohen's *d*, Pearson's *r*), indicating how they were calculated

Our web collection on statistics for biologists contains articles on many of the points above.

Software and code

Policy information about availability of computer code

Data collection	MRI data was acquired with a 3T Siemens MR scanner (Erlangen, Germany).
Data analysis	MRI data was analysed with Matlab version 2018a (Mathworks, Natick, MA, USA). Regions of interest were drawn with MIPAV (https://mipav.cit.nih.gov/). Statistical anlaysis was performed with IBM SPSS Statistics (version 25, Chicago, IL, USA)

For manuscripts utilizing custom algorithms or software that are central to the research but not yet described in published literature, software must be made available to editors and reviewers. We strongly encourage code deposition in a community repository (e.g. GitHub). See the Nature Portfolio guidelines for submitting code & software for further information.

Data

Policy information about availability of data

All manuscripts must include a data availability statement. This statement should provide the following information, where applicable:

- Accession codes, unique identifiers, or web links for publicly available datasets
- A description of any restrictions on data availability
- For clinical datasets or third party data, please ensure that the statement adheres to our policy

Source data for the figures are available as Supplementary Data 1. All post-processed data generated or analysed during this study are included in Supplementary Data 2. All other data is available from the corresponding author upon reasonable request.

Human research participants

Policy information about studies involving human research participants and Sex and Gender in Research.

Reporting on sex and gender	We described the sex (biological attribute) of our research participants. We did not study differences between sex because of the limited sample size. Information about gender (shaped by social and cultural circumstances) has not been collected.
Population characteristics	We included 9 patients (7 male and 2 female) diagnosed with FSHD type 1 and with an average age of 61 years (range: 46–72 years). Seven of these patients (7 male) underwent a follow-up MRI scan 3 years and 8 months after the baseline scan (range: 3 years and 5 months to 3 years and 10 months).
Recruitment	The FSHD patients were recruited via the local Neurology clinic.
Ethics oversight	The study was approved by the local medical ethics committee CMO Arnhem-Nijmegen and all subjects gave written informed consent.

Note that full information on the approval of the study protocol must also be provided in the manuscript.

Field-specific reporting

Please select the one below that is the best fit for your research. If you are not sure, read the appropriate sections before making your selection.

☒ Life sciences ☐ Behavioural & social sciences ☐ Ecological, evolutionary & environmental sciences

For a reference copy of the document with all sections, see nature.com/documents/nr-reporting-summary-flat.pdf

Life sciences study design

All studies must disclose on these points even when the disclosure is negative.

Sample size	We analyzed data collected from 9 participants
Data exclusions	In one participants, data from ten lower leg muscles were excluded from further analysis because the Dixon fat-water reconstruction for the baseline MRIs resulted in a fat-water swap in the middle of one lower leg.
Replication	Reproducibility was not assessed in this study
Randomization	Randomization was not performed because this is a natural history study
Blinding	Blinding was not performed, because this is a natural history study and all participants have FSHD

Reporting for specific materials, systems and methods

We require information from authors about some types of materials, experimental systems and methods used in many studies. Here, indicate whether each material, system or method listed is relevant to your study. If you are not sure if a list item applies to your research, read the appropriate section before selecting a response.

Materials & experimental systems

n/a	Involved in the study	
☒	☐	Antibodies
☒	☐	Eukaryotic cell lines
☒	☐	Palaeontology and archaeology
☒	☐	Animals and other organisms
☐	☒	Clinical data
☒	☐	Dual use research of concern

Methods

n/a	Involved in the study	
☒	☐	ChIP-seq
☒	☐	Flow cytometry
☒	☐	MRI-based neuroimaging

Clinical data

Policy information about clinical studies

All manuscripts should comply with the ICMJE guidelines for publication of clinical research and a completed CONSORT checklist must be included with all submissions.

Clinical trial registration	N/A, not an interventional study
Study protocol	N/A, not an interventional study
Data collection	All data was collected Radboud university medical centre. Patient were recruited from 2014 to 2015. Data collection was performed between from 2014 to 2018.
Outcomes	Outcome measure were change in fat fraction from baseline to follow-up at the subject level and for specific muscle groups. Furthermore, we assessed how fat infiltration was distributed along the proximo-distal axis of individual muscles and how this distribution altered over time.
